# Supplementary material for: Human Neural Stem Cell-Based Drug Product: Clinical and Nonclinical Characterization
Source: Int J Mol Sci. 2022 Nov 3;23(21):13425. doi: 10.3390/ijms232113425 (PMC9653902; doi:10.3390/ijms232113425)
Supplement: Supplementary file 1 [file ijms-23-13425-s001.zip › ijms-1964530-supplementary.pdf]

Supplementary materials for:
Human neural stem cell-based drug products: clinical and non-clinical characterization

Par. 2.1: Identity - Positionl identity

Table S1: Raw data on real time PCR for the two samples and the total normal human brain (NHB) used as positive control.

Cell Line	Detector	Mean Ct	StdDev Qty	Delta Ct
05/12 B	*gapdh*	14,930	0,085	
	EmxI	36,655	0,177	21,725
	FoxgI	16,690	0,184	1,760
	Gsh2	19,680	1,202	4,750
	Nkx2.1	30,940	0,891	16,010
	Dlx2	28,045	0,332	13,115
	Emx2	22,765	0,021	7,835
	Irx3	29,660	0,198	14,730
	En2	35,125	0,460	20,195
	Gbx2	22,290	1,075	7,360
	Hoxb1	40,000	0,000	25,070
	Hoxb5	40,000	0,000	25,070
08/12B	*gapdh*	18,030	0,311	
	Emx1	38,750	1,768	20,720
	Foxg1	19,230	0,042	1,200
	Gsh2	23,945	0,262	5,915
	Nkx2.1	40,000	0,000	21,970
	Dlx2	31,780	0,311	13,750
	Emx2	25,015	0,035	6,985
	Irx3	28,965	0,035	10,935
	En2	36,295	0,134	18,265
	Gbx2	26,420	0,057	8,390
	Hoxb1	40,000	0,000	21,970
	Hoxb5	40,000	0,000	21,970
NHB	*gapdh*	15,645	0,035	
	EmxI	22,835	0,714	7,185
	FoxgI	18,580	0,057	2,930
	Gsh2	28,000	0,014	12,350
	Nkx2.1	28,080	1,004	12,430
	Dlx2	21,775	0,530	6,125
	Emx2	22,340	0,255	6,690
	Irx3	25,645	0,318	9,995
	En2	23,525	0,233	7,875
	Gbx2	28,330	0,368	12,680
	Hoxb1	40,000	0,000	24,350
	Hoxb5	32,105	0,332	16,455

Supplementary materials for:
Human neural stem cell-based drug products: clinical and non-clinical characterization

Par. 2.1: Identity - In vitro differentiation test

Table S2: For each sample, using a fluorescence microscope and a 20X lens, we counted at least 2000 cells in not less than 5 fields. Then we determined the percentage of each cell type on the total number of counted differentiated cells. Here are reported the calculated percentages of neurons, astrocytes and oligodendrocytes in each batch, the average value for alle the batches and the standard deviation.

BATCH #	% Neurons	% Astrocytes	% Oligodendrocytes
1	30	49	21
2	31	52	18
3	31	49	29
4	33	50	18
5	24	54	23
6	29	49	21
7	20	65	15
8	48	41	12
9	30	52	18
10	17	59	24
11	40	47	13
12	28	46	25
13	32	53	15
14	36	46	18
15	43	45	11
16	25	28	36
17	46	26	26
18	17	12	26
19	27	26	19
20	16	40	7
21	13	20	11
22	7	46	36
23	18	48	29
24	16	41	24
25	26	54	18
26	15	55	31
27	22	53	31
28	10	37	11
29	8	40	9
30	10	55	29

Average	24,91	44,57	20,75
ST.DEV.	11,14	11,99	7,98

Supplementary materials for:
Human neural stem cell-based drug products: clinical and non-clinical characterization

Par. 2.3.3 Safety - in vivo test

Table S3: Here we present raw data of the in vivo safety evaluation for the two donors we used in the ALS trial. The hNCSCs lines were unilaterally transplanted into the dorsal striatum (300.000 cells/mice) of Athymic Nude mice and animals were kept for up to 6 months (n=7 animals for B05/12 and n=8 animals for B08/12). See main article for protocol description.

Cell line	Number of cells	Tumors formation	% of survived cells [1]	Ki67 [2]	Stem cells [3]	Astrocytes [4]	Neuronal progenitors [5]	Neurons [6]	Oligodendrocytes [7]	Migration into the brain parenchima (µm)
B05/12	300.000	no	32,98± 4,39	4,83±1,12	20,7±3,37	40,88±5,96	12,39±3,04	11,83±1,83	12,8±0,48	6150 ± 584,05
B08/12	300.000	no	36,78 ± 7,71	3,98±0,72	35,42±2,80	27,29±1,47	6,95±0,72	8,62±0,71	4,44±0,50	4714,29 ± 491,84

[1] % of survived cells respect to the total injected
[2] %Ki67/tot huN
[3] %Nestin+/tot huN
[4] %GFAP+/tot huN
[5] %Dcx+/tot huN
[6] %b-Tub III+/tot huN
[7] %MBP+/tot huN
